# Optimizing High-Intensity Functional Training Performance: Individualized Load Prescription vs. Standardized Weights

**DOI:** 10.3390/sports14030108

**Published:** 2026-03-09

**Authors:** Alejandro Oliver-López, Rafael Sabido, Tom Brandt, Annette Schmidt

**Affiliations:** 1 Sport Research Center, Miguel Hernandez University of Elche, 03202 Elche, Spain; 2 Department of Sports Sciences, Faculty of Medicine, Health and Sports, Universidad Europea de Madrid, 28670 Madrid, Spain; rsabido@umh.es; 3 Institute of Sports Science, University of the Bundeswehr Munich, 85579 Neubiberg, Germanyannette.schmidt@unibw.de (A.S.)

**Keywords:** functional fitness training, individualization, adaptation, performance

## Abstract

Background: This study compares the effects of relativized barbell loads (% of one-repetition maximum or 1RM) versus standardized prescribed loads on High-Intensity Functional Training (HIFT) performance, strength adaptations, physiological response, and perceived effort. Methods: In total, 22 experienced HIFT athletes (12 males, 10 females) were randomly assigned to either a standardized load (SL) or relativized load (RL) group. Both groups completed an 8-week HIFT program with benchmark workouts. Performance was assessed using a local muscle endurance test, maximal strength through 1RM testing (back squat, clean, and clean and jerk), and neuromuscular performance via countermovement jump (CMJ). Cardiopulmonary response (VO^2^peak, VO^2^mean, heart rate, and blood lactate levels) and perceived effort (Borg CR-10) were also evaluated. Results: RL participants did not show a difference in the interaction between group and time in TT performance but differences were founded for strength gains in back squat (*p* = 0.005, 95% CI [3.1, 8.6]) and clean (*p* = 0.027, 95% CI [1.2, 5.7]) compared to the SL group. No significant differences were found in clean and jerk performance or CMJ height. Cardiopulmonary responses were similar between groups, indicating comparable physiological stress. RL participants reported significantly lower perceived exertion (*p* < 0.001, 95% CI [6.3, 9.8]), suggesting more efficient load management and recovery. Conclusions: Use of individualized loads based on 1RM enhanced HIFT performance and strength adaptations, without increasing physiological stress, enabling more efficient training with reduced fatigue.

## 1. Introduction

Unlike High-Intensity Interval Training (HIIT), which is primarily based on cyclical and repetitive exercises performed over predefined work–rest intervals; HIFT emphasizes varied, non-cyclical, multi-joint functional movements, often integrating strength, power, and metabolic conditioning within a single training session [1]. The use of HIFT-based exercise programs has expanded globally, attracting a wide-range of users, and its physiological and psychological effects have been investigated [2,3]. In fact, among overweight adults, students, diabetics, and sedentary workers, HIFT-based interventions have been shown to improve body composition, fitness, insulin sensitivity, and exercise program adherence [4,5,6,7].

In HIFT gyms (“boxes”), competitive athletes can train alongside individuals aiming to improve their health [8]. Coaches prescribe standard workouts, such as benchmark WODs, using fixed barbell weights, or “scaled” WODs with simplified movements or reduced weights in an attempt to adapt the workouts to wider audiences [9]. However, scaled WODs do not incorporate individualized training loads, potentially limiting progress and increasing the risk for injury [10].

Individualization of training loads for HIFT-based exercise programs can be challenging, because they involve a mix of gymnastics, endurance, and strength training [11]. Nevertheless, strength training is traditionally prescribed using one-repetition maximum (1RM), the gold standard for training prescription and assessment [12]. HIFT sessions often include a strength component (e.g., back squat [BS], clean [C], or clean and jerk [CJ]), where intensity is determined based on a percentage of the measured 1RM. However, WODs prescribe fixed loads that are predefined by gender, regardless of individual strength levels.

In the context of HIFT, only one study has compared the effects of using standardized loads (50 kg for males, 35 kg for females in CJ) versus relativized loads set at 75% of 1RM for a specific WOD [10]. The results showed that relativized loads led to more homogeneous repetitions and a greater physiological impact with respect to blood lactate [La], countermovement jump [CMJ], heart rate [HR], and perceived effort [RPE] among stronger athletes.

Because of the above, the aim of the present study was to compare changes in Tibana Test (TT) performance, 1RM, CMJ height, VO_2_, HR, [La], and RPE following HIFT interventions with standardized (SL) vs. relativized (RL) loads. We hypothesize that RL will lead to greater improvements in TT, 1RM, and CMJ, while both intervention methods will result in efficient physiological adaptations.

## 2. Materials and Methods

### 2.1. Overview of the Experimental Design

In the present study, an experimental intervention was conducted on experienced HIFT athletes randomly assigned to two groups, SL and RL, which utilized fixed or individualized loads during HIFT workouts, respectively. Interventions were conducted from May to July 2024, in two HIFT boxes in Spain, while data was collected in the laboratory. The interventions were incorporated into the athletes’ existing periodized training plans by replacing specific scheduled training components, thereby preserving their habitual routines and minimizing interference with their ongoing training progress. Group progress was evaluated at the start, middle (only 1RM assessment), and end of the study.

Before enrollment, study participants were informed about the protocol and potential risks and provided written consent. The study followed the Declaration of Helsinki and was approved by the ethics committee of Miguel Hernández University (Ethics Committee ID: 230210121042).

### 2.2. Subjects

The sample size was estimated based on a previous systematic review of HIFT training responses [2]. Using G*Power Version 3.1.9.6, a sample size of 16 participants was required for an ANOVA with a power of 0.8 and an alpha level set at 0.05 [13]. Twenty-five participants were recruited at the start of this intervention. However, 3 participants dropped out during the study period: one participant withdrew from the study because of personal circumstances, while the other two participants withdrew because of knee and lower back pain and discomfort. An injury rate of 8% was observed during the present study. In total, 22 participants (12 males and 10 females) completed the HIFT training intervention (Table 1).

All study participants were required to meet the following inclusion criteria: 12 months of HIFT experience; ability to perform the minimum required gymnastic movements; and ability to lift standardized weights in overhead movements. In addition, study exclusion criteria were as follows: (i) use of performance-enhancing drugs; (ii) pregnancy; or (iii) the presence of health issues or injuries that would affect HIFT training.

During the intervention, participants were instructed to refrain from outside exercise and maintained their usual diet. Two intervention groups were defined: (i) SL, which utilized standardized loads for males and females; and (ii) RL, which used relativized loads calculated based on the percentage of 1RM. As shown in Table 1, both intervention groups were balanced for strength ratios and gender [10].

### 2.3. Design

Before beginning the HIFT training intervention, study participants were evaluated at our laboratory at the start of the first week, in order to obtain the following descriptive measurements: height, body weight, and HIFT experience. In addition, TT analysis was performed, coupled with simultaneous assessment of cardiopulmonary variables (VO_2_ and HR). During this week, and at least 72 h after the laboratory measurements, participants were required to determine their 1RM for BS, CJ, and C in their HIFT box, following the protocol reported by Baechle and Earle [14].

**Figure 1:**
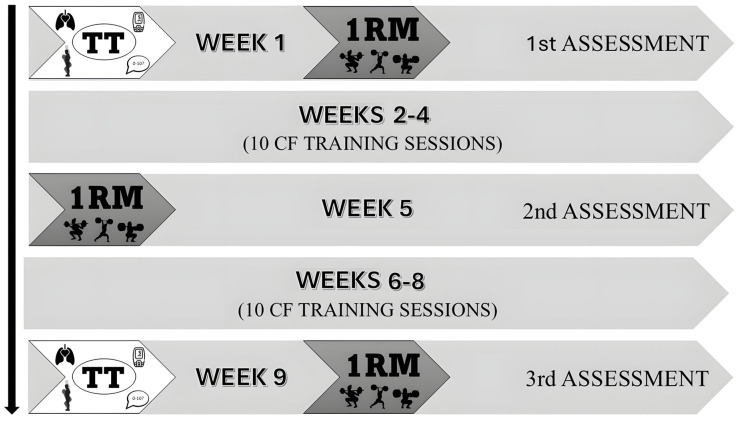
Study development and preliminary assessment points. TT (Tibana Test). 1RM: Estimation of the one repetition maximum for the back squat, clean and jerk, and clean/TT: Tibana Test, which measures performance in terms of number of repetitions, ventilatory response, heart rate, blood lactate, CMJ, and perceived effort.

The HIFT training intervention comprised 10 benchmark WODs during the second, third, and fourth weeks followed by a repeat of these WODs during the sixth, seventh, and eighth weeks. In the fifth week, participants only had to perform three 1RMs, in order to adjust barbell weights for the RL group (Figure 1). All HIFT sessions included a general warm-up, specific WOD preparation, the HIFT benchmark WOD, and accessory work, totaling one hour per session. A comprehensive list of all the HIFT training sessions can be found in Supplementary Table S1.

Moreover, to adjust barbell weights for participants in the SL group, we followed the standard weights for males and females in benchmark WODs during all the interventions, but for the RL group we selected and changed the weights according to the steps below:

1. A literature review established the average 1RM for experienced HIFT athletes for the BS, CJ, and C. Reported values for the CJ were 100, 93, and 101 kg for males and 64, 60, and 68 kg for females, leading to reference averages of 101 kg (males) and 64 kg (females) [15,16]. These values were then used to calculate the relative load intensities for benchmark WODs in terms of %1RM: for example, a WOD prescribing 60 kg for males and 40 kg for females in the CJ represents approximately 60% of their 1RM.
2. Correlations and regression models were used to adjust barbell loads across different movements using the BS, CJ, and C as predictors [16,17,18]. Weightlifting manuals, such as Everett’s [19], were used to provide empirical relationships for load prescriptions, without requiring complete 1RM data: for example, the prediction equation developed by Oliver-López et al. [18] was used to estimate the thruster maximum load based on the CJ 1RM.
3. In WODs involving multiple barbell movements, a single load was set based on the overhead movement, since this typically involves lifting less weight than floor-based movements, such as deadlifts or snatches: for example, in the DT workout, the intensity was prescribed using the CJ 1RM, since the push jerk determines the limiting load. Literature-based relationships were applied when the overhead movement differed from the push jerk kinematics; thus, in the “Dany” workout, the push press load was set at ~88% of the split jerk [17].

A weight calculator for the WODs used in this study can be found in the Supplementary Materials.

### 2.4. Methodology

Study participants completed a WOD in laboratory conditions pre- and post-HIFT intervention. One day before, they were advised not to exercise vigorously or consume large amounts of alcohol or caffeine. Three hours prior, they were instructed to have a healthy meal and hydrate. Before the TT, all participants warmed up by rowing at an easy pace for 3 min, followed by two sets of 10 alternating lunges, 20 shoulder taps in plank position, and 10 s in an isometric push-up position. After this, participants rowed for 2 min at medium intensity. The WOD used was an adapted version of the TT [20] composed of four rounds of As Many Repetitions As Possible (AMRAPs), where subjects were required to perform the maximum number of repetitions in each AMRAP for a duration of 4 min, with 2 min of passive rest after every workout. This modified version was used due to laboratory constraints that made it infeasible to include all of the original elements.

**Figure 2:**
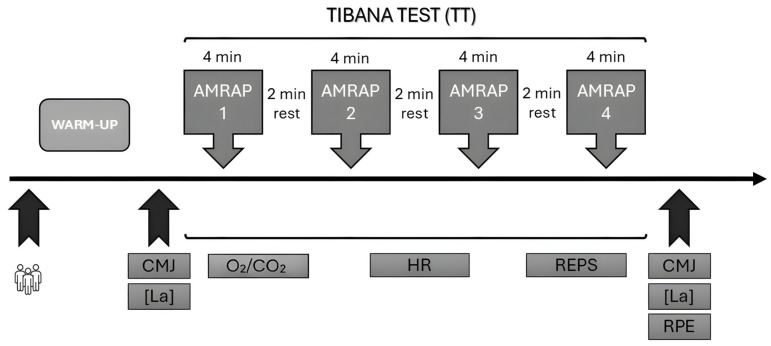
Timeline of assessments of study participants during testing days. CMJ (countermovement jump); [La] (blood lactate); O^2^/CO^2^ (respiratory exchange); Reps (repetitions); RPE (Rate of Perceived Effort). The following acronyms were used in this figure. WHO-5: well-being questionnaire/CMJ: maximum height in countermovement jump/[La]: blood lactate/RPE: Rate of Perceived Effort 0-10/O^2^/CO^2^: ventilatory exchange/HR: heart rate as the mean and maximum/REPS: Performance measure of the number of repetitions during the test.

The TT workout was divided into: (round 1) a 4 min AMRAP of five thrusters with two dumbbells (17.5 kg and 12.5 kg; male and female weights, respectively) and five box steps (50/40 cm; male and female height, respectively) with dumbbells; (round 2) a 4 min AMRAP of 10 power cleans with dumbbells and 10 rows with dumbbells; (round 3) a 4 min AMRAP of 15 shoulders to overhead with dumbbells and 30 V sit-ups; and (round 4) a 4 min AMRAP of 20 calories of rowing on a Concept II indoor rowing machine (PM5, Morrisville, VT, USA) and 40 squat jumps. The TT protocol and all explained measurements are shown in Figure 2.

#### 2.4.1. Performance

In order to guarantee that the prescribed movement, workout, and training requirements were fulfilled during the TT, each AMRAP of the test was supervised by one certified individual who had completed the ‘CrossFit Judges’ Course. To score valid repetitions for each participant during the assessment, the researchers relied on a description of the knowledge and abilities required, and competition-like movement criteria were followed, including identification of common faults. During all measurement sessions, verbal encouragement was used, and repetitions were counted aloud, to motivate participants to give their best effort [21]. Researchers recorded the total score for all four AMRAPs and the number of repetitions for each AMRAP.

#### 2.4.2. One-Repetition Maximum

For each participant, 1RMs were assessed for the BS, CJ, and C movements, which were performed according to Baechle and Earle’s guidelines [14]. The time points for these measurements were before the start of the HIFT intervention, at the middle of the intervention during the fifth week, and at the end of the study in the ninth week. These three 1RMs were estimated during the same session in the following order: (1) BS, (2) CJ, and (3) the maximum load in the C movement. Finally, 5 min of rest between attempts was given between the BS and the CJ, and between the CJ and the C [22].

#### 2.4.3. Performance in CMJ

Jump height was estimated using a contact platform and Chronopic hardware (Chronojump, Boscosystem, Barcelona, Spain). A specific warm-up of six progressive CMJs every 30 s was performed before recording the best jump out of three attempts at the end of the warm-up, and immediately after completion of the WOD test [23,24]. In addition, the best CMJ tested after the warm-up was used to compare neuromuscular differences between the start and end of the intervention. Participants had to keep their hands on their hips during all trials and land with their knees straight. Rebound-on landing was allowed by flexing the ankles. Upon first contact with the mat, the stopwatch was stopped, and only the best jump was considered for investigation due to potential learning effects, and to avoid greater jump performances [25].

#### 2.4.4. Cardiopulmonary Exercise Testing (CPET)

Before starting the test, a special face mask was worn by the study participants, and the last part of the warm-up was performed wearing this face mask to familiarize them with the mask. During all AMRAPs, ventilatory exchange was measured with a portable Breath-by-Breath system (MetaLyzer 3B-R3, CORTEX, Leipzig, Germany). To smooth data from the gas analyzer, the signal was averaged every 10 s. Both the maximum oxygen peak (VO^2^peak) and average oxygen consumption (VO^2^mean) were calculated for posterior analysis and were expressed relative to participant body weight (mL/min/kg) [26]. Heart rate was continuously monitored using a Bluetooth heart rate belt located in the Xiphoid area. Peak heart rate (HRpeak) and average heart rate (HRmean) were assessed for posterior analysis (Polar H9, Polar, Kempele, Finland). Finally, a total record of ventilatory exchange and heart rate was obtained for 25 min, including the tests’ AMRAPs, rest time, and three minutes after the test, to observe the recovery phase.

#### 2.4.5. Analysis of Blood Lactate Levels

Blood lactate levels [La] were determined from peripheral blood samples taken from the left earlobe using test strips and a portable analyzer device (Lactate Scout Plus, EKF Diagnostics, Leipzig, Germany). Before collection, the skin was wiped with chlorhexidine. The first two drops of blood were discarded, and the third was used for analysis. Lactate levels were measured at one minute after warm-up (basal) and at the end of the WOD test after the CMJ [27].

#### 2.4.6. Rate of Perceived Exertion

The Rate of Perceived Exertion (RPE) was determined using the Borg category scale (CR-10), and all participants were familiarized with this perceived exertion scale before the start of the study [28]. The CR-10 scale ranks exercise intensity from 0–10, where 0 corresponds to “rest”, and 10 corresponds to “maximum” intensity. Participants were asked “How hard do you think the WOD was?” at the end of each AMRAP, and 30 min after the test WOD, to ensure that perceived exertion referred to the whole workout rather than the most recent exercise intensity.

#### 2.4.7. Training Load During the Intervention

The training load for the study participants was estimated using two methods. The first method only considered the total time required by the participants to complete the workouts. The average time for each WOD and the total time of the intervention were calculated. In contrast, the second method used the total number of repetitions performed by each participant multiplied by the barbell weight load used during the workout. The average load for each WOD and the total load for the entire HIFT training were calculated [29].

### 2.5. Statistical Analysis

Performance variables for the four AMRAPs (total repetitions, VO_2_peak, VO_2_mean, HRpeak, HRmean, [La], CMJ, and RPE) were analyzed using a factorial ANOVA with two levels (pre and post) and two factors (SL and RL), with the exception of 1RM in BS, CJ, and C, which had three time points (pre, mid, post). Absolute and relative changes in [La] and CMJ before and after TT were calculated for pre–post comparisons. Data was tested for independence, homoscedasticity, and normality using Durbin–Watson, Levene, and Shapiro–Wilk tests. Means and standard deviations (M ± SD) were reported, and the effect size (ES) was calculated using omega squared (ω^2^) with thresholds for small (0.01 ≤ ω^2^ < 0.06), medium (0.06 ≤ ω^2^ < 0.14), and large (ω^2^ ≥ 0.14) effects [30]. Tukey’s correction and Cohen’s d were used for post hoc comparisons. Performance changes in 1RM, CMJ, and TT repetitions were analyzed individually using the Smallest Worthwhile Change (SWC), with a threshold of 0.2 for trained populations [31,32]. The sample was divided into responders and non-responders based on SWC, and the responder rate was expressed as a percentage per group. Differences in total and mean training time and load between groups were assessed with an independent *t*-test, and similar analyses were performed for each WOD at different time points. Statistical significance was set at *p* < 0.05, and analyses were conducted using JASP (v0.18.3.0).

## 3. Results

The primary finding of the present study was that only the RL group displayed a significant improvement in their total performance in the TT test after the HIFT intervention, as demonstrated by a significant interaction effect (ES = 0.54, *p* = 0.009). In contrast, the SL group showed a non-significant trend toward improvement (ES = 0.44, *p* = 0.085). Moreover, both groups showed performance improvements in the four AMRAPs of the TT from pre- to post-intervention (ES = 0.51, *p* = 0.001), but with no significant interaction.

**Figure 3:**
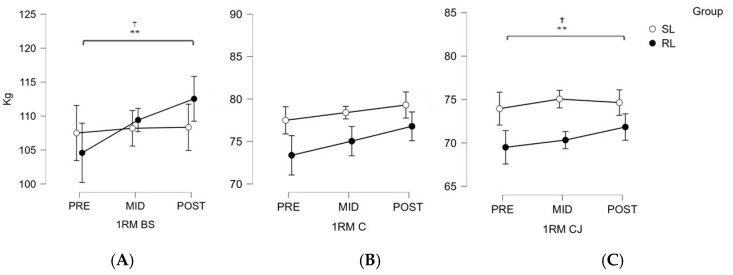
Graphical representation of the three 1RM assessments of the back squat, the clean and jerk, and the clean conducted pre-, mid- and post-intervention. BS (back squat); C (clean); CJ (clean and jerk); SL (standardized load); RL (relativized load). Note. (**A**) 1RM for the back squat; (**B**) 1RM for the clean and jerk; (**C**) 1RM for the clean. SL = standardized group, represented by open circles; 1RM = One repetition maximum; RL = relativized group, represented by solid black circles; PRE = first week of the intervention; MID = fifth week of the intervention; POST = ninth week of the intervention (the final week). ** *p* < 0.01; ^†^ differences between PRE and POST in RL group.

With respect to strength adaptations, participants in the RL group displayed a significant increased in their 1RMs for the BS (ES = 0.31, *p* = 0.005) and the C (ES = 0.16, *p* = 0.027). Meanwhile, no significant 1RM changes were observed for these parameters in the SL group after HIFT training (Figure 3A,B). In both groups, no significant changes in CJ performance were observed.

With respect to neuromuscular adaptations, neither the RL nor the SL group significantly improved their CMJ height scores by the end of the intervention. In addition, based on the TT, a significant decrease in CMJ height was measured immediately after the TT for the RL and SL groups, both before (SL: ES = 0.83, *p* < 0.001; RL: ES = 0.7, *p* < 0.001) and after the intervention (SL: ES = 0.57, *p* < 0.001; RL: ES = 0.55, *p* < 0.001). However, no significant interaction effects were observed between groups or across time points (Table 2).

Cardiopulmonary variables were derived from CPET analysis (VO^2^peak, VO^2^mean, HRpeak, and HRmean) and showed no significant changes pre- and post-intervention. Similarly, as shown in Table 2, no significant interaction effects were found for blood lactate levels between time points or between groups.

Perceived effort remained similar between groups pre- and post-intervention. However, at the first assessment, the RL group reported a lower RPE than the SL group, which had a significantly higher final RPE at the end of the TT (ES = 0.59, *p* < 0.001).

**Figure 4:**
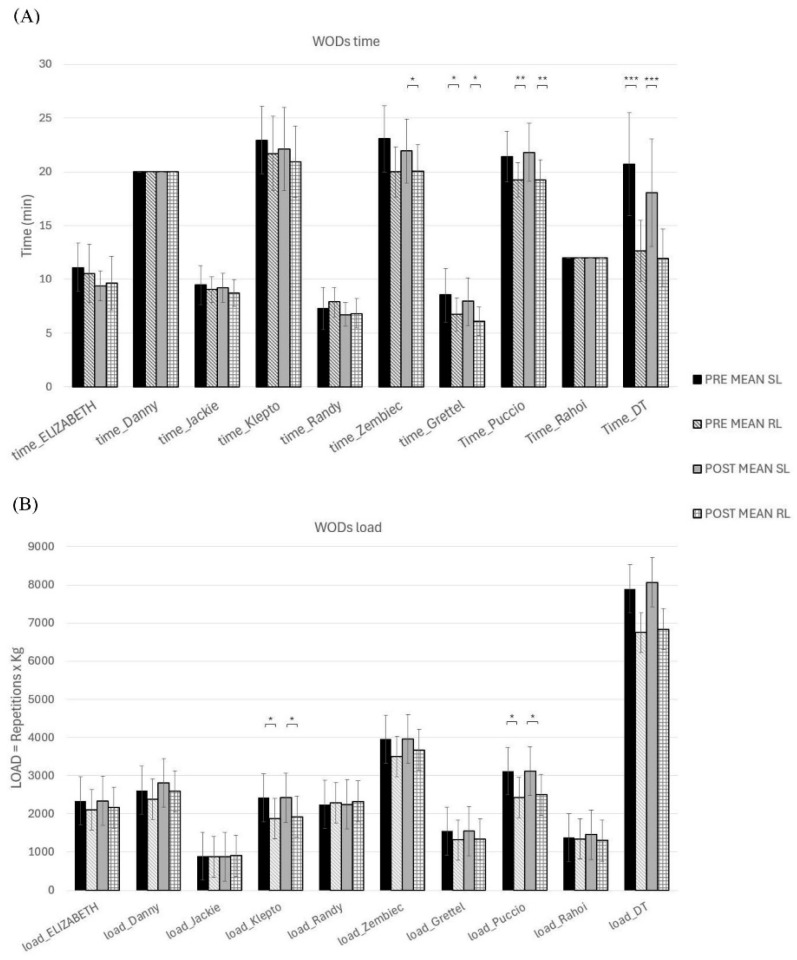
Comparison of training loads during the intervention for athletes from the SL and RL groups. (**A**) Average training time; (**B**) Average training load (repetitions x kg barbell weight). The bars indicate the first or second WOD and the intervention group (SL or RL). Note: * *p* < 0.05; ** *p* < 0.01; *** *p* < 0.001.

Based on training load analysis, no significant differences in total training time or total load were identified between the RL and SL groups during the intervention. However, based on analyses of each benchmark WOD, the SL group spent significantly more time in the first Zembiec WOD (ES = 0.55, *p* = 0.004) and the first and second Grettel (ES = 0.49, *p* = 0.017; ES = 0.51, *p* = 0.01), Puccio (ES = 0.52, *p* = 0.008; ES = 0.54, *p* = 0.005), and DT workouts (ES = 0.68, *p* < 0.001; ES = 0.61, *p* < 0.001). In addition, the SL group lifted a significantly greater total load during the first and second Klepto (ES = 0.51, *p* = 0.011; ES = 0.49, *p* < 0.017), Puccio (ES = 0.51, *p* = 0.01; ES = 0.48, *p* < 0.018) and DT WODs (ES = 0.37, *p* < 0.001; ES = 0.42, *p* < 0.001) compared to the RL group (Figure 4).

**Figure 5:**
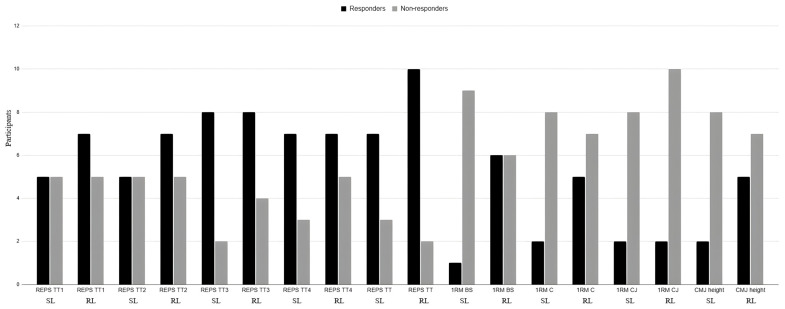
Individual statistics for responders, non-responders and negative responders with respect to 1RM performance, CMJ height and Tibana Test performance by group (SL or RL) using the Smallest Worthwhile Change (SWC).

Finally, we found that 70% of the participants in the SL group were classified as responders. In addition, over 83% of RL group members had a positive result, and were also classified as responders to the training intervention. However, this difference was not statistically significant. Moreover, with respect to 1RM improvements, fewer than 20% of SL participants showed clear individual progress in the BS and C categories, while approximately 50% of RL participants improved in both exercises. A similar trend was observed for CMJ height, where approximately 20% of SL participants were classified as responders, compared to over 45% from the RL group (Figure 5).

## 4. Discussion

In the present study, the effects of an HIFT intervention were evaluated on a population of experienced athletes by comparing changes in workout performance, strength gains, jump height, and physiological responses between a group using individualized loads (RL) and a group using prescribed Rx loads (SL). Although the participants in the RL improved significantly with respect to TT performance, no interaction between group and time was identified. Strength gains in terms of 1RM for BS and C were greater in the RL group, but no improvements in CMJ height were observed for either group. No significant differences between groups were identified with respect to VO_2_, HR, or [La] values before and after TT, despite the fact that high acute neuromuscular and physiological responses were observed.

Although only the RL group demonstrated statistically significant within-group improvements in TT performance, the absence of consistent interaction effects on secondary variables suggests that conclusions regarding superior performance should be interpreted cautiously. Previous studies have shown that adjusting exercise intensity based on HR and HRV can improve performance and fatigue management. DeBlauw reported a 12.21% improvement in AMRAP repetitions with HRV-based programming versus pre-established protocols (+14.7%) [33]. Similarly, over a 6-week period, polarized HIFT training produced similar improvements in WOD (+10.7% vs. +9.9%) as high-intensity training [34]. These findings highlight the benefits of tailored load management.

Regarding strength, RL improved 1RM in BS and C, while no improvements were observed in the SL group. This is in agreement with Posnakidis, who found greater 1RM increases with higher loads (>80% 1RM) versus moderate loads (~60% 1RM) in HIFT (+19.2% vs. +10.4% in bench press) [35]. RL participants trained at ~60% 1RM, while SL participants used variable loads, reducing training consistency. Another study showed 4.7% and 4.5% increases in squat and deadlift 1RM after eight weeks of HIFT, despite the fact that only the strength portion was individualized, and not the WOD itself [36]. This suggests that load individualization during the WOD may further enhance strength adaptations.

CMJ height did not improve significantly in either group. This contrasts with previous HIFT studies showing increases of between 6.2% and 8% in CMJ after 6 to 8 weeks [35,37]. However, in the present study, the relatively high training experience (≥12 months) of the participants may have reduced sensitivity to neuromuscular adaptation. Additionally, HIFT prioritizes efficiency over maximal jump height, for example by focusing on fast box jumps, which could explain the lack of CMJ improvement despite the incorporation of power-based exercises in the WODs.

No significant differences were found in VO^2^, HR, or [La] before and after the intervention. This is in agreement with reported findings that HIFT induces rapid muscle fatigue before the VO^2^peak is reached, limiting aerobic capacity improvements. Rios described VO^2^ kinetics in HIFT as a rapid phase followed by a slower phase, which may explain our observation of stable VO^2^ values. Held [34] reported no VO^2^peak differences after six weeks of high-intensity training, suggesting improved efficiency rather than increased aerobic capacity. However, studies on untrained participants have shown VO^2^max improvements of +6.4% to +10.4% following HIFT interventions [3,38].

RPE levels recorded after TT (7.7–8.6 on the Borg CR10) confirmed that the HIFT workouts were conducted at high intensity [2]. Only the SL group showed a significant post-intervention increase (+10.5%), indicating better fatigue management in the RL group due to load individualization. RL participants reported a mean RPE of 7.3 ± 0.91 versus 7.7 ± 0.85 in SL. Despite high perceived effort, feedback from HIFT athletes has been positive, and they often report enjoyment and program adherence due to the social environment [4].

Members of the SL group trained 10.6% longer than RL group participants, but no significant differences were found in total load or training time across WODs. However, SL participants trained longer and lifted more weight in certain WODs. Similar findings have been reported with HR-based training, where lower loads produced comparable performance gains [34]. Similarly to our study, in this HR-based training, no injuries were observed in the group that individualized the WODs intensities. Thus, in both HR-based and barbell (load-based) training, no injuries occurred in the RL group. In contrast, injuries did occur in the groups that did not relativize (individualize) this variable, and two participants were not able to complete the intervention due to injuries. However, HIFT programs combining strength training, gymnastics, and endurance introduce heterogeneity in the exercise prescription, making it challenging to understand which factors should be considered when prescribing WODs (e.g., exercise volume, intensity, and duration) and which method HIFT coaches should use to prescribe and quantify the load for their athletes in order to achieve the desired adaptations and manage fatigue [39].

Moreover, individual SWC analysis, which was conducted to monitor participant improvements in the specific study variables, revealed a higher number of positive responders in the RL group (50–60% positive responders) than in the SL group. Few studies have examined load individualization in the context of HIFT, but general sports science supports tailored intensity to optimize adaptation [40]. Some systematic reviews of HIFT [2,38] recommend individualized training with defined goals and periodicity due to improper load adjustments that can lead to detrimental adaptations to training.

The present study has several limitations. First, the relatively small sample size (*n* = 22) may have limited the statistical power to detect group × time interaction effects, increasing the possibility of type II error in several secondary outcomes. Second, the mixed-sex composition of the sample (12 males and 10 females), although balanced between groups, may have introduced additional variability in strength and physiological responses. The study was not powered to conduct sex-specific analyses. Third, while a significant interaction effect was observed for total TT performance, no significant interaction effects were detected for several secondary variables (CMJ, VO_2_, HR, lactate, and individual AMRAPs). Therefore, interpretations regarding the superiority of relativized loading should be limited primarily to the primary performance outcome. Fourth, the absence of a non-training control group limits the ability to isolate the effect of load prescription from general training adaptations. Finally, the intervention duration may not have been sufficient to detect longer-term neuromuscular or aerobic adaptations in experienced athletes.

### Practical Applications

Based on the present findings, we recommend prescribing loads for HIFT exercise programs based on individualized 1RM percentages determined for each athlete, and not as a function of Rx or scale-established loads. The process involves (1) determining reference Rx loads for key movements (BS, CJ, C) based on the literature, and calculating %1RM for WODs; (2) estimating unknown 1RMs using regression models; and (3) adjusting loads for multiple barbell movements based on overhead lifts to maintain workout intensity.

## 5. Conclusions

The use of individualized loads was associated with greater improvements in TT performance and certain strength measures compared to the use of standardized barbell loads. However, given the sample size and the absence of significant interaction effects across several secondary variables, these findings should be interpreted cautiously. Further research is needed to confirm whether relativized load prescription consistently produces superior adaptations in HIFT athletes.

## Figures and Tables

**Table 1:** Descriptive statistics of the 22 study participants at baseline.

	All Participants (*n* = 22)	Standardized Load Group (*n* = 10)	Relativized Load Group (*n* = 12)
Male (*n*)	12	5	7
Female (*n*)	10	5	5
Age (y)	38.1 ± 7.8	36.8 ± 8.5	39.3 ± 7.4
Height (cm)	171.2 ± 8.9	171.6 ± 10.4	170.9 ± 8.1
Weight (kg)	73.8 ± 11.3	73.8 ± 9.5	73.8 ± 12.9
Ratio ^1^	1.4 ± 0.2	1.4 ± 0.3	1.4 ± 0.2
CF experience (months)	77.5 ± 36.3	82.9 ± 36.8	73.0 ± 36.9

^1^ maximal weight lifted in back squat divided by body weight.

**Table 2:** Changes in measured Tibana Test parameters, shown as the mean ± SD for participants from the standardized and relativized groups for the abovementioned study variables.

	SL (*n* = 10)			RL (*n* = 12)			*p*-Value Time Effect	*p*-Value Group x Time Effect
	Pre (M ± SD)	Post (M ± SD)	*p*-Value	Pre (M ± SD)	Post (M ± SD)	*p*-Value		
AM1 (reps)	60.1 ± 8.9	62.6 ± 5.2	0.511	56 ± 8	59.3 ± 7.5	0.203	0.025	0.733
AM2 (reps)	70 ± 10.2	74.1 ± 11.7	0.282	66.8 ± 7.2	69.8 ± 10.5	0.491	0.030	0.698
AM3 (reps)	81.1 ± 18.4	85.7 ± 15.9	0.601	81.3 ± 15.4	90.1 ± 11.9	0.073	0.014	0.413
AM4 (reps)	79.7 ± 16.1	84.8 ± 19.3	0.184	74.3 ± 9.8	79.3 ± 15.4	0.141	0.006	0.976
WOD (reps)	290.9 ± 41.1	307.2 ± 45	0.063	278.4 ± 27.3	298.4 ± 35.4 *	0.009	0.001	0.657
VO^2^peak (#)	41.1 ± 5.5	40.2 ± 5.8	0.804	41.9 ± 3.8	40.2 ± 2.9	0.251	0.064	0.536
VO^2^mean (#)	29 ± 2.6	28.8 ± 3.9	0.995	29.6 ± 2.4	29.2 ± 3.3	0.948	0.595	0.852
HRpeak (bpm)	174 ± 5.4	172.9 ± 5.2	0.695	176.5 ± 6.1	174.3 ± 6.6	0.137	0.028	0.444
HRmean (bpm)	157.1 ± 6.5	155.7 ± 5.5	0.783	157.7 ± 4.8	154.9 ± 6.1	0.227	0.026	0.498
∆[La] (mmol/L)	12.2 ± 1.6	13.8 ± 1.8	0.084	12.7 ± 2.7	13.9 ± 2	0.175	0.003	0.669
∆CMJ (%/cm)	−17.2 ± 6.2	−13.5 ± 7.9	0.577	−14.2 ± 10.6	−11.9 ± 9.1	0.756	0.120	0.776
RPE (0/10 au)	8.2 ± 0.4	8.6 ± 0.7	0.406	7.7 ± 0.7	7.9 ± 0.7	0.701	0.001	0.233

Note. SL = standardized group; RL = relativized group; AM = As Many Repetitions as Possible; reps = repetitions; WOD: workout of the day; # = mL/min/kg; bpm = beats per minute; ∆[La] = percent change in blood lactate levels; mmol = millimoles per liter; ∆% CMJ = percent change in countermovement jump; au = arbitrary units * *p* < 0.05.

## Data Availability

No new data were created or analyzed in this study.

## Supplementary Materials

The following supporting information can be downloaded at: https://www.mdpi.com/article/10.3390/sports14030108/s1, Table S1: The 8-week CF-intervention list with the measures and WODs.

## Abbreviations

The following abbreviations are used in this manuscript:

HIFT	High-Intensity Functional Training
WOD	Workout of the day
TT	Tibana Test
CMJ	Countermovement jump
[La]	Blood lactate
RPE	Rating of perceived effort
SL	Standardized load
RL	Relativized load
SWC	Smallest Worthwhile Change
BS	Back squat
CJ	Clean and jerk
C	Clean
