# Supplementary material for: Optimizing High-Intensity Functional Training Performance: Individualized Load Prescription vs. Standardized Weights
Source: Sports (Basel). 2026 Mar 9;14(3):108. doi: 10.3390/sports14030108 (PMC13030661; doi:10.3390/sports14030108)
Supplement: Supplementary file 1 [file sports-14-00108-s001.zip › sports-4081098-supplementary.pdf]

Table S1. The 8-week CF-intervention list with the measures and WODs.

WEEK / DAY SESSION	Measure	Warm-Up	RL WOD	SL WOD	Accessory
**W1/Monday**	Tibana Test (repetitions, VO2, blood lactate, CMJ, RPE)	- 5´ row easy pace // 2RD of: - 8 squats - 6 lunges - 10 shoulder taps - 10´´ iso push	TIBANA TEST (2DB 17,5/12,5 Kg) 2´ rest between AMRAPs <u>a/AMRAP 4’</u>: 5 Thrusters 5 Step Box <u>b/AMRAP 4’</u>: 10 Power Clean 10 DB Row	<u>c/AMRAP 4’</u>: 15 Shoulder to OH 30 V Sit Ups <u>d/AMRAP 4’:</u> 20 cal. Row erg 40 squats jump	10´ bike erg easy pace
**W1/Thursday**	1RM (Back Squat, Clean & Jerk and Clean)	Mobility and stability (2x10): - 90/90º hip - Bleeding down to lunge - Ankle dorsiflexion - Airplane Shoulder mobility (2x10) - “YTWL” - Shoulder taps	A) 1RM BACK SQUAT - 4x50% (1´) - 3x75% (1´30") - 2x85% (3') - 1x93% (3') - 1xRM (3') - Search for PR B) 1RM CLEAN & JERK “The same 1RM protocol (28)” B.1) Record best CLEAN at the end of the maximum weight lift in Clean & Jerk		10´ Foam Roller
**W2 & W6/ Monday**	RPE post-WOD	5´ erg easy pace // 3RD of: - Push Ups to bear - Lunge with thoracic rotation, - Pull Down // Specific WOD warm-up	ELIZABETH (21.15.9 F.T.) - Power Cleans (64% 1RM Clean & Jerk) - Ring Dips	ELIZABETH (21.15.9 F.T.) - Power Cleans (61/43 Kg) - Ring Dips	10´ Pull up skill
**W2 & W6/ Thursday**	RPE post-WOD	5´ mobility with stick // Specific WOD warm-up: 3RD of: - 6 Box Step over - 4 Push press - 5 Pull-ups	DANNY (AMRAP 20´) - 30 Box Jump (60/50 cm) - 20 Push Press (59% 1RM Clean & Jerk) - 30 Pull Ups	DANNY (AMRAP 20´) - 30 Box Jump (60/50 cm) - 20 Push Press (52/34 Kg) - 30 Pull Ups	10´ Seated stretching
**W2 & W6/ Friday**	RPE post-WOD	10´ barbell warm-up (3x5): deadlift, shoulder press, and back squat // Specific WOD warm-up: 3RD of: - 200m row erg - 4 Thrusters - 5 Pull band	JACKIE (F.T.) - 1000 m row - 50 Thrusters (24% 1RM Clean & Jerk) - 30 Pull Ups	JACKIE (F.T.) - 1000 m row - 50 Thrusters (20/15 Kg) - 30 Pull Ups	8´ Tabata - 10m shuttle run - Burpees to target - Jumping Jacks - Hollow Rocks

WEEK / DAY SESSION	Measure	Warm-Up	RL WOD	SL WOD	Accessory
**W3 & W7/ Monday**	RPE post-WOD	10´ EMOM<br>- 1ST erg<br>- 2ND dynamic mobility // Specific WOD warm-up<br>4RD of:<br>- 4 plyometric box jumps<br>- 4 Worms to push up<br>- 2 Power Cleans	KLEPTO<br>(4RD F.T.)<br>- 27 Box Jumps (60/50 cm)<br>- 20 Burpees<br>- 11 Squat Cleans (58%1RM Clean & Jerk)	KLEPTO<br>(4RD F.T.)<br>- 27 Box Jumps (60/50 cm)<br>- 20 Burpees<br>- 11 Squat Cleans (65/45 Kg)	10´ bike erg easy pace
**W3 & W7/ Tuesday**	RPE post-WOD	5´ mobility with stick // Specific WOD warm-up<br>3RD of:<br>- 1 Muscle Snatch<br>- 1 Snatch balance<br>- 2 OHS<br>- 1 power snatch	RANDY (F.T.)<br>-75 Power Snatches (55%1RM Clean & Jerk)	RANDY (F.T.)<br>-75 Power Snatches (35/25 Kg)	15´ Ring Skill
**W3 & W7/ Thursday**	RPE post-WOD	5´ mobility and activation (2x10) Lunge and thoracic mobility, push up to bear and pull over // Specific WOD warm-up<br>3RD of:<br>- 5 Globet squat<br>- 4 Burpees to target<br>- 4 Pull-ups<br>- 4 Shuttle run	ZEMBIEC<br>(5RD F.T.)<br>- 11 Back Squats (61% 1RM Back Squat)<br>- 7 Burpees Pull Up<br>- 400m run	ZEMBIEC<br>(5RD F.T.)<br>- 11 Back Squats (83/61 Kg)<br>- 7 Burpees Pull Up<br>- 400m run	5´ Foam Roller
**W3 & W7/ Friday**	RPE post-WOD	5´ mobility work (2x10)<br>90/90 hip rotations, shoulder taps, and pike push-ups // Specific WOD warm-up<br>3RD of:<br>- 1 High Pull<br>- 1 Power Clean<br>- 1 Front Squat<br>- 1 Split Jerk	GRETTEL<br>(10RD F.T.)<br>- 3 Clean & Jerks (63% 1RM Clean & Jerk)<br>- 3 Bar Over Burpees	GRETTEL<br>(10RD F.T.)<br>- 3 Clean & Jerks (60/43 Kg)<br>- 3 Bar Over Burpees	10´CORE work (3x10)<br>- Press pallof<br>- Rotation trunk<br>- Pallof and overhead

WEEK / DAY SESSION	Measure	Warm-Up	RL WOD	SL WOD	Accessory
**W4 & W8/ Monday**	RPE post-WOD	5´ hip and ankle mobility (3x10) 90/90 hip rotations, ankle dorsiflexion // Specific WOD warm-up<br>3RD of:<br>- 8 row cal.<br>- 1 Power Clean<br>- 2 Back squats<br>- 100m run	PUCCIO (F.T.)<br>- 1000 m row<br>- 30 Power Cleans (60% Clean & Jerk)<br>- 30 Back Squats (55% 1RM Back Squat)<br>- 1.6 Km run	PUCCIO (F.T.)<br>- 1000 m row<br>- 30 Power Cleans (61/43 Kg)<br>- 30 Back Squats (61/43 Kg)<br>- 1.6 Km run	10´ FOAM & stretching
**W4 & W8/ Thursday**	RPE post-WOD	5´ KB activation (3x10)<br>KB around the hip, lateral stand-up, and unilateral shoulder press // Specific WOD warm-up:<br>3RD of:<br>- 4 Box step over<br>- 3 Thruster<br>- 2 Burpees	RAHOI (AMRAP 12')<br>- 12 Box Jump (60/50 cm)<br>- 6 Thrusters (49% 1RM Clean & Jerk)<br>- 6 Bar Facing Burpees	RAHOI (AMRAP 12')<br>- 12 Box Jump (60/50 cm)<br>- 6 Thrusters (43/30 Kg)<br>- 6 Bar Facing Burpees	10´ CORE work<br>- Frontal plank<br>- Lateral plank<br>- Unilateral bridge
**W4 & W8/ Friday**	RPE post-WOD	5´ mobility with stick // Specific WOD warm-up<br>3RD of:<br>- 4 Deadlifts<br>- 1 Squat cleans<br>- 2 Push Jerks	DT (5RD F.T)<br>-12 Deadlifts (72% 1RM Clean & Jerk)<br>- 9 Hang Power Cleans<br>- 6 Push Jerks	DT (5RD F.T)<br>- 12 Deadlifts (70/47.5 Kg)<br>- 9 Hang Power Cleans<br>- 6 Push Jerks	10´ Plyometric upper body technique
**WEEK / DAY SESSION**	**Measure**	**Warm-Up**	**RL WOD**	**SL WOD**	**Accessory**
**W5/Monday**	1RM (Back Squat, Clean & Jerk and Clean)	"	"	"	"
**W9/Monday**	Tibana Test (repetitions, VO2, blood lactate, CMJ, RPE)	"	"	"	"
**W9/Thursday**	1RM (Back Squat, Clean & Jerk and Clean)	"	"	"	"

*W = week; RD = round; PR = personal record; DB = dumbbell; KB = kettle bell; OH = overhead; cal. = calories; erg = ergometer; WOD = workouts of the day; F.T. = for time
